# Facial Skin Aging Stages in Chinese Females

**DOI:** 10.3389/fmed.2022.870926

**Published:** 2022-04-27

**Authors:** Xiao-xiao Yang, Meng-meng Zhao, Yi-fan He, Hong Meng, Qing-yang Meng, Qiao-yin Shi, Fan Yi

**Affiliations:** ^1^Key Laboratory of Cosmetic, China National Light Industry, College of Chemistry and Materials Science, Beijing Technology and Business University, Beijing, China; ^2^Institute of Cosmetic Regulatory Science, College of Chemistry and Materials Science, Beijing Technology and Business University, Beijing, China; ^3^Shanghai Pechoin Daily Chemical Co., Ltd., Shanghai, China

**Keywords:** facial skin, skin aging, non-invasive evaluation, Chinese female, individual skin care

## Abstract

**Background:**

Facial skin is exposed to the environment, which marks it with obvious signs of aging. Based on multi-dimensional non-invasive evaluation data, female facial skin can be characterized in detail. However, there are few studies on the general aging rules of facial skin. Most skin aging studies divide the ages into 5–10-year intervals, so they have lacked dynamic matching with facial skin aging.

**Aim:**

To explore facial skin aging rules, discuss the main parameters of facial skin aging, propose an unequal-distance aging division method based on the main skin parameters, and study the skin characteristics of Chinese women of different aging stages.

**Methods:**

We comprehensively described the skin status as 24 non-invasive skin parameters belonging to five dimensions: skin wrinkles, texture, stain, color and barrier function. We performed polynomial fitting on the 21 skin parameters that were significantly correlated with age and derived the rules of aging in the different dimensions. Based on the wrinkle dimension, the facial skin aging process was divided into four stages, and the skin characteristics of the different stages were compared.

**Results:**

Skin wrinkles increased, texture deteriorated, acne decreased, pigment spots increased, skin tone darkened, and sebum secretion decreased with age, according to the polynomial fitting. The aging stage was divided into an incubation period (18–30 years old), an aging occurrence period (31–42 years old), a rapid aging period (43–47 years old), and a stable aging period (48–60 years old), according to the wrinkles. Different aging stages had different skin characteristics.

**Conclusion:**

The incubation period is the critical period for the appearance of skin stains; the skin texture gradually deteriorates during the aging occurrence period; the rapid aging period is a critical period for the aging of skin parameters; skin status during the stable aging period is the worst.

## Introduction

Skin aging is a complex biological behavior (1) involving changes in multiple skin physiological parameters (2) and appearance characteristics (3) and has attracted considerable attention from researchers in many disciplines. It consists of two independent processes: endogenous aging is mainly controlled by genes (4), and exogenous aging is mainly affected by solar radiation (5). Histological studies have shown that endogenous aging causes the epidermis to thin, shrink, display fine lines and dry out. Exogenous aging causes the skin epidermis to thicken, loosen, deepen wrinkles, stain, and become duller and rougher (6, 7).

Facial skin is the skin most prone to aging-related changes in humans (8). The skin gradually ages due to the effects of internal and external factors, resulting in visible changes in facial skin morphology, especially wrinkles and sagging (9). Due to the complexity of skin aging, it is necessary to comprehensively characterize the state of aging skin from multiple dimensions. As a highly dynamic organ, skin aging process can be divided into several stages that comply with the rules of dynamic aging. However, current research mainly focuses on the anatomic, cellular and molecular mechanisms of Caucasians. There are few papers on the aging rules of Chinese female facial skin, and the dimensions in which skin conditions can be characterized need to be expanded. In addition, there is no unanimous conclusion about the skin aging stages of Chinese women.

To non-invasively evaluate facial skin, a multifunctional skin physiology monitor (Courage & Khazaka Electronic GmbH, Cologne, Germany) is commonly used because it can quantitatively determine skin parameters under constant temperature and humidity conditions, such as skin moisture content and sebum secretion (10). The emergence of multi-light-source skin photography technology has provided a quantitative basis for the description of facial skin problems, such as the number of acne marks. A commonly used instrument is the VISIA-CR (Canfield Scientific, Parsippany-Troy Hills, NJ, United States) (11). So far, most descriptions of skin problems are based on the subjective judgmentof researchers or dermatologists, which have limited objectivity and reproducibility. By applying a machine learning algorithm, we extracted the cross-polarized light images of volunteers taken by the VISIA-CR in batches, which we used to objectively, non-invasively, quantitatively classify skin parameters.

**FIGURE 1:**
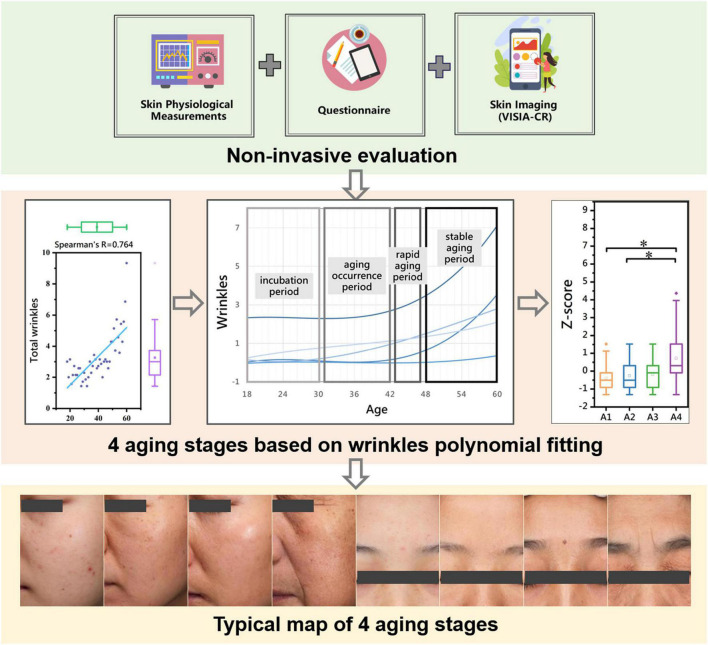
Graphical overview of the facial skin aging study in Chinese female population aged 18–60. *P < 0.05 between the two aging stages.

We collected 24 skin parameters of 300 Chinese female volunteers aged 18–60 based on non-invasive skin tests (done by the multifunctional skin physiology monitor and the VISIA-CR) to characterize the skin status of Chinese women of different ages. Then, correlation analysis and polynomial fitting between skin parameters and age were performed to derive the aging rules of facial skin parameters with age. The main parameters of facial skin aging are discussed, and an unequal-distance aging division method with age based on the main parameters is proposed (Figure 1). This study provides resources for research and development on aging-related skin characteristics.

## Materials and Methods

### Subjects

The study was conducted in accordance with the principles of the Declaration of Helsinki. The study protocol was approved by an internal review board, and written informed consent to participate in the study was obtained from each volunteer. We conducted a randomized recruitment trial in five cities through a third-party testing institution (Eviskin cosmetics technology (Beijing) Co., Ltd., Beijing, China) in this study. The sample size of a sampling survey is affected by five factors: (a) acceptable precision/accuracy level; (b) sample idiosyncratic situation; (c) sample availability; (d) sampling technique used; (e) specific requirements of subsequent analysis methods. Taking five factors into consideration, the following formula is used for calculation: $\text{n}=\frac{z^{2}\cdot p\cdot \left(1-p\right)}{d^{2}}$ (12, 13). Among them, the *z*-value represents the confidence level, and *z* = 1.96 corresponding to the 95% confidence level is selected; Since there is no previous data, the *p*-value is set to 0.5; the *d-*value is acceptable precision/accuracy level, usually taken as 0.05. Thus, the sample size calculation result of this survey is 266.78. Considering that the design of the follow-up experimental method is 43 age groups between 18 and 60 years old, the number of study subjects is uniformly distributed, so a total of 300 Chinese female volunteers aged 18–60 years (mean 38.93 ± 12.39 SD) were enrolled. Each age included 7 volunteers, except only 6 aged 60. The exclusion criteria were as follows: (a) current pregnancy or menstruation; (b) infectious skin disease; (c) had an anti-immunologic therapy within the last 3 months; (d) used steroid drugs within 1 month; and (e) facial surgery, including medical beautification, within 6 months.

### Non-invasive Evaluation Design

All subjects had lived in Beijing (north latitude 39°56′, east longitude 116°20′), Shanghai (north latitude 31°22′, east longitude 121°48′), Wuxi (north latitude 31°57′, east longitude 120°30′), Wuhan (north latitude 30°52′, east longitude 114°31′) or Taiyuan (north latitude 37°87′, east longitude 112°53′) for at least 1 year. The measurements were performed from August to December 2019. Volunteers were allowed to relax in a room with a temperature of 22 ± 2°C and a relative humidity of 50 ± 5% for 30 min after washing their face with a cleanser.

### Instrumental Measurements and Skin Parameters

Measurements were taken with the VISIA-CR and various probes attached to a multifunctional skin physiology monitor (Courage + Khazaka Electronic GmbH, Cologne, Germany). The 11 skin biophysical parameters (CM, TEWL, SM, pH, MEXA, ERYTH, b*, ITA°, GLOSS_DSC, Ra and R2) were measured at three anatomical sites on the face: forehead, left cheek, left canthus. VISIA-CR images (front and left face) were taken to extract 13 skin parameters (total wrinkles, eyebrow lines, crow’s feet, fine lines around the eyes, perioral lines, total stains, freckles, sunburn, age spots, acne, acne marks, pores, and blackheads) through machine learning algorithms. The meanings of the specific skin parameters and the equipment that measured them are given in Table 1.

**TABLE 1:** Skin parameters and test equipment for non-invasive evaluation.

Parameter dimension	Parameter name	Parameter meaning	Test equipment and specifications
Facial skin barrier dimension	CM	Skin moisture content	Corneometer CM 825
	TEWL	Transdermal water loss	Tewameter TM300
	SM	Sebum secretion	Sebumeter SM815
	pH	Skin pH	Skin-pH-Meter PH905
Facial skin tone dimension	MEXA	Melanin content	Mexameter MX18
	ERYTH	Haem content	Mexameter MX18
	b*	Skin yellowness	Colorimeter CL400
	ITA°	Individual type angle	Colorimeter CL400
	GLOSS_DSC	Skin gloss	GL200
Facial skin texture dimension	R2	Skin elasticity	Cutometer dual MPA580
	Ra	Arithmetic mean roughness	PRIMOS/Evaskin
	Acne	Number of acnes	VISIA-CR
	Acne marks	Number of acne marks	VISIA-CR
	Blackheads	Number of blackheads	VISIA-CR
	Pores	Number of pores	VISIA-CR
Facial stain dimension	Total stains	Total number of stains	VISIA-CR
	Freckles	Number of freckles	VISIA-CR
	Sunburns	Number of sunburns	VISIA-CR
	Age spots	Number of age spots	VISIA-CR
Facial wrinkle dimension	Total wrinkles	Total number of wrinkles	VISIA-CR
	Eyebrow lines	Number of lines between eyebrows	VISIA-CR
	Fine lines around eyes	Number of fine lines around eyes	VISIA-CR
	Crow’s feet	Number of crow’s feet	VISIA-CR
	Perioral lines	Number of lines around mouth	VISIA-CR

### Statistical Analysis

SPSS Statistics 25.0 (IBM, New York, NY, United States) and MATLAB Starter Application R2020a (MathWorks. Inc., Massachusetts, CA, United States) were used for statistical analysis. All data are expressed as mean ± SD (standard deviation). Spearman’s correlation coefficients between skin parameters and age were calculated (14). The curve and formula of skin parameters with age were obtained by polynomial fitting, and the inflection point and stationary point were calculated to accurately derive the aging rules. The significant difference in skin parameters between aging stages was calculated by one-way ANOVA with Bonferroni’s post-test (15). The statistical tests were two-tailed with significance levels of 0.05.

## Results

We comprehensively considered the above two factors by measuring parameters in five dimensions (skin barrier, color, texture, stains, and wrinkles) to characterize the skin status of the Chinese female volunteers and performed descriptive statistics on their skin parameters (Table 2).

**TABLE 2:** Descriptive statistics of skin parameters and their correlation with age.

Parameter dimension	Skin parameter	(Min, max)	Mean ± SD	Correlation coefficient	*P*-value (Sig.)
Facial skin barrier dimension	CM	(20.8, 90.42)	60.36 ± 11.49	0.068	0.666
	TEWL	(7.5, 28.03)	15.79 ± 4.16	−0.493**	0.001
	SM	(0, 144.86)	42.29 ± 31.68	−0.867**	0.000
	pH	(4.67, 7.15)	6.01 ± 0.44	0.164	0.292
Facial skin tone dimension	MEXA	(56.9, 237)	142.63 ± 31.83	0.568**	0.000
	ERYTH	(181.2, 526.67)	325.15 ± 58.35	0.008	0.960
	b*	(6.24, 9.34)	12.95 ± 2.62	−0.902**	0.000
	ITA°	(19.33, 62.17)	40.62 ± 8.9	0.828**	0.000
	GLOSS_DSC	(1.39, 8.11)	4.2 ± 1.24	0.503**	0.001
Facial skin texture dimension	R2	(0.36, 0.88)	0.61 ± 0.1	−0.532**	0.000
	Ra	(0.01, 0.09)	0.02 ± 0.01	0.634**	0.000
	Acne	(0, 96)	13.77 ± 9.63	−0.512**	0.000
	Acne marks	(0, 24)	6.36 ± 5.09	−0.831**	0.000
	Blackheads	(0, 29)	6.84 ± 6.27	0.721**	0.000
	Pores	(0, 176)	47.12 ± 36.31	0.634**	0.000
Facial stain dimension	Total stains	(7, 215)	89.00 ± 44.51	0.655**	0.000
	Freckles	(5, 214)	85.81 ± 43.00	0.618**	0.000
	Sunburns	(0, 52)	2.86 ± 4.59	0.708**	0.000
	Age spots	(0, 8)	0.32 ± 1.06	0.827**	0.000
Facial wrinkle dimension	Total wrinkles	(0, 14)	3.24 ± 2.46	0.764**	0.000
	Eyebrow lines	(0, 7)	0.63 ± 1.43	0.751**	0.000
	Fine lines around eyes	(0, 3)	0.04 ± 0.27	0.435**	0.004
	Crow’s feet	(0, 7)	0.97 ± 1.22	0.914**	0.000
	Perioral lines	(0, 4)	1.07 ± 1.00	0.843**	0.000

*P < 0.05, **P < 0.01, correlation between skin parameters and age.

### Correlation Between Skin Parameters and Age

**FIGURE 2:**
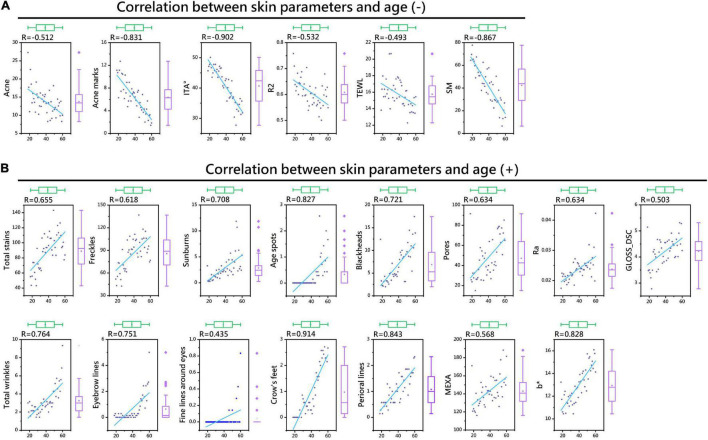
Thermal map of the correlation coefficients between skin parameters and age. Skin parameters negatively correlated with age **(A)**. Skin parameters positively correlated with age **(B)**.

Spearman correlation analysis was used to measure the relationships between 24 skin parameters and age. The results showed the following: (a) Except for CM, pH, and ERYTH (*P* > 0.05), all the skin parameters were correlated with age; (b) TEWL, SM, acne, acne marks, ITA°, and R2 were negatively correlated with age (*P* < 0.01) (Figure 2A and Table 2); (c) MEXA, b*, GLOSS_DSC, Ra, blackheads, pores, total wrinkles, eyebrow lines, fine lines around the eyes, crow’s feet, perioral lines, total stains, freckles, sunburn, and age spots were positively correlated with age (*P* < 0.01) (Figure 2B and Table 2). Therefore, polynomial fitting models of 21 skin parameters with age could be established.

### Skin Parameter-Age Polynomial Fitting Models

The polynomial fitting models of 20 skin parameters and age were established. The fitting curve formula is shown in Table 3.

**TABLE 3:** Inflection points and stationary points of skin parameters based on polynomial fitting.

Skin parameter	Polynomial fitting curve formula	Stationary point 1	Stationary point 2	Inflection point
Total wrinkles	*f*_*TW*_(*x*) = 0.0001352*x*^3^−0.01068*x*^2^ + 0.2712*x* + 0.1146	21.3613	31.3014	26.3314
Eyebrow lines	*f*_*EL*_(*x*) = 0.0001482*x*^3^−0.01337*x*^2^ + 0.3857*x*−3.456	24.0055	36.1385	30.072
Fine lines around eyes	*f*_*FL*_(*x*) = 0.00002764*x*^3^−0.00284*x*^2^ + 0.09259*x*−0.9496	26.7375	41.7623	34.2499
Crow’s feet	*f*_*CF*_(*x*) = −0.00002792*x*^3^ + 0.004999*x*^2^−0.1865*x* + 2.032	23.1394	96.2254	59.6824
Perioral lines	*f*_*PL*_(*x*) = 0.00003081*x*^3^−0.003278*x*^2^ + 0.146*x*−1.509	–	–	35.4647
R2	*f*_*R*2_(*x*) = 0.000001739*x*^3^−0.00006185*x*^2^−0.005867*x* + 0.8063	−23.7134	47.4243	11.8555
Ra	*f*_*Ra*_(*x*) = 0.0000004386*x*^3^−0.00004842*x*^2^ + 0.001855*x*−0.001362	–	–	36.7989
Acne	*f*_*A*_(*x*) = −0.0005298*x*^3^ + 0.06741*x*^2^−2.857*x* + 53.26	41.2881	43.5364	42.4122
Acne marks	*f*_*AM*_(*x*) = −0.0001441*x*^3^ + 0.01685*x*^2^−0.8035*x* + 20.6	–	–	38.9776
Blackheads	*f*_*BH*_(*x*) = −0.0002073*x*^3^ + 0.03228*x*^2^−1.295*x* + 19.32	27.1699	76.641	51.9055
Pores	*f*_*P*_(*x*) = −0.001265*x*^3^ + 0.19*x*^2^−7.765*x* + 129.7	28.6071	71.5246	50.0659
Total stains	*f*_*TS*_(*x*) = −0.002859*x*^3^ + 0.2672*x*^2^−5.792*x* + 88.32	13.9711	48.3351	31.1531
Freckles	*f*_*F*_(*x*) = −0.002534*x*^3^ + 0.2272*x*^2^−4.389*x* + 72.35	12.1139	47.6598	29.8869
Sunburns	*f*_*S*_(*x*) = −0.000315*x*^3^ + 0.03751*x*^2^−1.283*x* + 14.45	24.9326	54.4536	39.6931
Age spots	*f*_*AS*_(*x*) = −0.000009078*x*^3^ + 0.002513*x*^2^−0.1205*x* + 1.52	28.3217	156.227	92.2744
MEXA	*f*_*M*_(*x*) = 0.0001623*x*^3^ + 0.0001347*x*^2^−0.05713*x* + 132.5	−11.1123	10.559	−0.2766
b*	*f*_*b*_(*x*) = −0.000197*x*^3^ + 0.02533*x*^2^−0.9183*x* + 21.56	26.033	59.6862	42.8596
ITA°	*f*_*ITA*_(*x*) = 0.0006319*x*^3^−0.07765*x*^2^−2.585*x* + 21.06	23.2354	58.6868	40.9611
GLOSS_DSC	*f*_*G*_(*x*) = −0.0000126*x*^3^ + 0.00142*x*^2^−0.02532*x* + 3.795	10.338	64.7943	37.5661
SM	*f*_*SM*_(*x*) = −0.0008836*x*^3^ + 0.1043*x*^2^−5.048*x* + 132.9	–	–	2.5083

#### Facial Wrinkle Dimension

**FIGURE 3:**
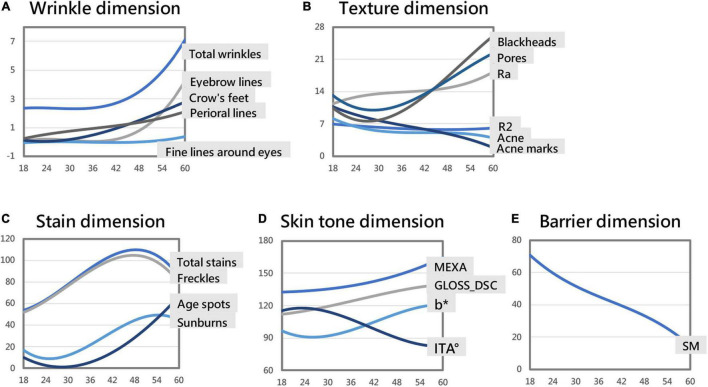
Skin parameter–age polynomial fitting curve models (some curves are in a consistent range through proportional scaling). Skin aging trend of the wrinkle dimension **(A)**. Skin aging trend of the texture dimension **(B)**. Skin aging trend of the stain dimension **(C)**. Skin aging trend of the skin tone dimension **(D)**. Skin aging trend of the barrier dimension **(E)**.

The skin parameters belonging to the wrinkle dimension showed an overall upward trend; crow’s feet and perioral lines increased with age, crow’s feet appeared at around 23 years old, and perioral lines increased at the fastest rate at around 35 years old. Total wrinkles, eyebrow lines, fine lines around the eyes increased significantly after the age of 42 (Figure 3A and Table 3).

#### Facial Skin Texture Dimension

With age, the number of blackheads and pores first fell and then rose. After 27 years old, the number of blackheads increased, the rate of increase peaking at around 52 years of age. After 29 years old, the number of pores increased, the rate of increase peaking around 50 years of age. The skin roughness Ra showed an upward trend, increasing fastest around 37 years old; the elasticity R2 showed a trend of first declining and then slowly rising, being smallest around 47 years old. The number of acne and acne marks decreased with age (Figure 3B and Table 3).

#### Facial Stain Dimension

As age increased, the total stains and freckles first increased and then decreased, reaching a maximum at about 48 years old. Age spots overall rose with age. After 28 years old, the number of age spots increased significantly. The number of sunburns first fell and then rose, being smallest around the age of 25 and then increasing. The growth rate of sunburns was the greatest around the age of 40, and the number of sunburns was the highest around the age of 54 (Figure 3C and Table 3).

#### Facial Skin Tone Dimension

The GLOSS_DSC value (skin glossiness) and the MEXA value (skin melanin content) showed an upward trend, increasing consistently with age. The skin yellowness b* value showed a trend of first declining and then increasing, being smallest around age 26 and then increasing. The growth rate of b* was the fastest around 43 years old, and the yellowest skin was around age 60. The ITA° value first rose and then fell. The skin tone was the lightest around the age of 23, and then the skin tone became darker. The skin tone darkening rate was the fastest around the age of 41, and the ITA° value of the skin around the age of 59 is the smallest, meaning the darkest skin (Figure 3D and Table 3).

#### Facial Skin Barrier Dimension

With age, the TEWL value of transcutaneous water loss showed a trend of rising first, then falling, and then slowly rising (almost unchanged). Peer et al. found that due to uncontrolled experimental variables, the repeatability of TEWL was difficult to guarantee (16), so we discarded this skin parameter. With age, the amount of sebum secretion SM showed a downward trend. Sebum secretion gradually decreased with age (Figure 3E and Table 3).

### Four Aging Stages According to the Key Points of Wrinkle Parameters

The key nodes of skin parameters were calculated by fitting curve formulas (Table 3). The stagnation point represents the turning point at which the trend of the function changes direction. The inflection point of the function is where the trend rate of the function changes. A skin parameter rises or falls fastest at its inflection point.

Spearman’s correlation coefficient showed that the skin parameters of the wrinkle dimension had the strongest correlation with age (Table 2). In addition, the main characteristics of facial skin aging are wrinkles and sagging (9). Compared with other skin parameters, wrinkles significantly affect appearance (17, 18). Therefore, based on the skin parameters of the wrinkle dimension (total wrinkles, brow lines, crow’s feet, fine lines around the eyes, perioral lines), we divided the skin aging of Chinese women into four stages: incubation period (18–30 years old), aging occurrence period (31–42 years old), rapid aging period (43–47 years old), and stable aging period (48–60 years old).

### Differences in Skin Parameters Between the Four Aging Stages

**FIGURE 4:**
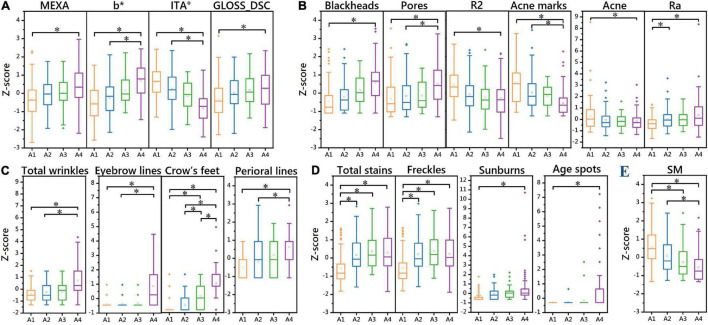
One-way ANOVA of 20 skin parameters in 4 aging stages (*Z* score). Differences in skin parameters of the skin tone dimension between the four aging stages **(A)**. Differences in skin parameters of the texture dimension between the four aging stages **(B)**. Differences in skin parameters of the winkle dimension between the four aging stages **(C)**. Differences in skin parameters of the stain dimension between the four aging stages **(D)**. Differences in skin parameters of the barrier dimension between the four aging stages **(E)**. *P < 0.05 between the two aging stages.

One-way ANOVA was performed on various skin parameters in the 4 aging stages. Because the data did not conform to a normal distribution, the Kruskal–Wallis test was selected to analyze their differences (Table 4). After Bonferroni correction, except CM, pH, ERYTH, crow’s feet, fine lines around the eyes, the other 20 skin parameters in the incubation period (A1), aging occurrence period (A2), rapid aging period (A3), and stable aging period (A4) had significant differences between the aging stages (Figure 4). The results of the pairwise rank sum test are shown in Table 5.

**TABLE 4:** Descriptive statistics and Kruskal–Wallis test of skin parameters between the four aging stages.

Skin parameter	Mean ± SD				*P*-value
	A1	A2	A3	A4	
Total wrinkles	2.26 ± 0.52	2.6 ± 0.6	2.74 ± 0.47	5.06 ± 1.65	0.000*
Eyebrow lines	0.09 ± 0.12	0.1 ± 0.11	0.17 ± 0.12	1.89 ± 1.18	0.000*
Fine lines around eyes	0 ± 0	0 ± 0	0.03 ± 0.06	0.13 ± 0.25	0.042*
Crow’s feet	0.15 ± 0.56	0.42 ± 0.76	1.14 ± 1.00	2.23 ± 1.06	0.000*
Perioral lines	0.56 ± 0.35	0.89 ± 0.37	1.26 ± 0.38	1.7 ± 0.41	0.000*
R2	0.65 ± 0.05	0.6 ± 0.04	0.58 ± 0.03	0.58 ± 0.05	0.002*
Ra	0.02 ± 0.00	0.02 ± 0.00	0.02 ± 0.00	0.03 ± 0.01	0.000*
Acne	16.57 ± 4.59	13.19 ± 2.45	12.34 ± 3.46	12.02 ± 2.59	0.020*
Acne marks	9.29 ± 2.12	6.51 ± 1.24	5.54 ± 1.96	3.57 ± 1.55	0.000*
Blackheads	3.97 ± 2.72	5.6 ± 1.8	7.8 ± 2.68	10.56 ± 3.82	0.000*
Pores	35.98 ± 19.74	41.31 ± 12.18	42.2 ± 14.42	65.7 ± 14.63	0.001*
Total stains	63.11 ± 13.64	96.82 ± 14.13	105.2 ± 23	101.37 ± 15.17	0.000*
Freckles	61.97 ± 13.54	94.42 ± 13.53	101.83 ± 21.85	95.45 ± 13.9	0.000*
Sunburns	1.14 ± 0.89	2.39 ± 0.8	3.23 ± 1.78	4.9 ± 3.2	0.000*
Age spots	0 ± 0	0.01 ± 0.04	0.14 ± 0.2	1.02 ± 0.72	0.000*
MEXA	132.97 ± 15.44	141.08 ± 8.99	143.38 ± 12.15	154.84 ± 18.55	0.008*
ERYTH	324.44 ± 21.1	326.34 ± 18.61	331.11 ± 23.13	321.21 ± 15.88	0.701
b*	11.58 ± 0.81	12.29 ± 1.03	13.36 ± 0.8	14.72 ± 1.02	0.000*
ITA°	46.34 ± 1.84	42.73 ± 3.42	39.47 ± 2.93	33.59 ± 2.79	0.000*
GLOSS_DSC	3.82 ± 0.65	4.2 ± 0.39	4.42 ± 0.37	4.53 ± 0.44	0.023*
CM	59.43 ± 2.99	59.43 ± 2.99	60.58 ± 3.62	59.84 ± 3.62	0.472
TEWL	16.58 ± 2.15	16.52 ± 1.07	15.14 ± 1	14.45 ± 1.16	0.001*
SM	60.27 ± 8.62	44.94 ± 10.13	33.8 ± 9.92	25.33 ± 11.76	0.000*
pH	6.00 ± 0.21	5.91 ± 0.12	5.93 ± 0.32	6.11 ± 0.19	0.086

*P < 0.05 between the four aging stages.

**TABLE 5:** Rank sum test of skin parameters in the four aging stages.

Skin parameter	*P*-value					
	A4-A3	A4-A2	A4-A1	A3-A2	A3-A1	A2-A1
Total wrinkles	0.069	0.001*	0.000*	1.000	1.000	1.000
Eyebrow lines	0.054	0.000*	0.000*	1.000	1.000	1.000
Fine lines around eyes	1.000	0.097	0.085	1.000	1.000	1.000
Crow’s feet	0.000*	0.000*	0.000*	0.002*	0.000*	0.356
Perioral lines	1.000	0.007*	0.000*	1.000	0.093	0.602
R2	1.000	1.000	0.004*	1.000	0.051	0.058
Ra	1.000	1.000	0.000*	1.000	0.217	0.025*
Acne	1.000	1.000	0.016*	1.000	0.334	0.387
Acne marks	0.740	0.020*	0.000*	1.000	0.113	0.180
Blackheads	1.000	0.053	0.000*	1.000	0.168	0.469
Pores	0.254	0.022*	0.000*	1.000	1.000	1.000
Total stains	1.000	1.000	0.000*	1.000	0.014*	0.002*
Freckles	1.000	1.000	0.000*	1.000	0.013*	0.001*
Sunburns	1.000	0.261	0.000*	1.000	0.097	0.085
Age spots	0.040*	0.000*	0.000*	1.000	1.000	1.000
MEXA	1.000	0.621	0.004*	1.000	1.000	0.492
b*	0.970	0.001*	0.000*	0.927	0.106	1.000
ITA°	0.531	0.002*	0.000*	1.000	0.086	0.384
GLOSS_DSC	1.000	1.000	0.018*	1.000	0.354	0.693
TEWL	1.000	0.003*	0.012*	0.374	0.746	1.000
SM	1.000	0.033*	0.000*	1.000	0.012*	0.114

*P < 0.05 between two aging stages.

#### Facial Wrinkle Dimension

There was no significant difference in fine lines around the eyes between the four aging stages. The results showed that there were significant differences in the parameters of total wrinkles, eyebrow lines, and perioral lines in the incubation period, aging occurrence period, and stable aging period (*P* < 0.05). Except for the comparison between the incubation period and the aging occurrence period, there were significant differences in crow’s feet between aging stages (*P* < 0.05). This indicates that the rapid aging period is a critical period for wrinkles, and the number of wrinkles increases significantly after the rapid aging period (Figure 4C and Table 5).

#### Facial Skin Texture Dimension

There were significant differences (*P* < 0.05) between the incubation period and the stable aging period in skin elasticity R2, total acne, and blackhead parameters. There were significant differences in the parameters acne marks and pores (*P* < 0.05) between the stable aging period and the incubation period and between the stable aging period and the aging occurrence period. There were significant differences in skin roughness Ra (*P* < 0.05) between the incubation period and the aging occurrence period and between the incubation period and the steady aging period.

This indicates that the aging occurrence period and the rapid aging period are critical periods for changes in skin elasticity, acne, and blackheads. After the aging occurrence period and the rapid aging period, the skin elasticity is significantly reduced, the number of acne is significantly reduced, and the number of blackheads is significantly increased. The incubation period is the critical period when the Ra parameter of skin roughness increases. The rapid aging period is a critical period when acne marks and pores change. After the rapid aging period, the number of acne marks is significantly reduced, and the number of pores is significantly increased (Figure 4B and Table 5).

#### Facial Skin Stain Dimension

The incubation period and other aging stages were significantly different in total stain and freckle parameters (*P* < 0.05); there were significant differences in sunburn parameters between the incubation period and the stable aging period (*P* < 0.05); the stable aging period was significantly different from the incubation period in age spots, as were other aging stages (*P* < 0.05). These results indicate that the incubation period is a critical period for the occurrence of stains, and the number of stains increases significantly after the incubation period. The rapid aging period is a critical period for the occurrence of senile plaques. After the rapid aging period, the number of senile plaques increases significantly (Figure 4D and Table 5).

#### Facial Skin Tone Dimension

The skin haem content (ERYTH) was not significantly different between the four aging stages. There were significant differences between the incubation period and the stable aging period in the skin melanin content (MEXA) and gloss (GLOSS_DSC) (*P* < 0.05); the skin yellowness b* value and skin color ITA° value parameters were significantly different (*P* < 0.05) Between the stable aging period and the incubation period, between the stable aging period and the aging occurrence period. This shows that the aging occurrence period and the rapid aging period are the key periods for skin color changes. After the aging period and the rapid aging period, the skin melanin content and gloss increase significantly, and the skin yellowness and skin color are significantly increased after the rapid aging period (Figure 4A and Table 5).

#### Facial Skin Barrier Dimension

There was no significant difference in the skin moisture content (CM) or pH between the four aging stages. TEWL was discarded. There were significant differences in sebum secretion (SM) (*P* < 0.05) between the aging occurrence period and the stable aging period, between the incubation period and the rapid aging period, and between the incubation period and the stable aging period (Figure 4E and Table 5).

## Discussion

### Multidimensional Description of Facial Skin Aging

The physiological state of women’s skin is closely related to skin health, and the skin aging process is often accompanied by changes in morphological characteristics. Taking the above two facts into consideration, we included skin barrier parameters (CM, TEWL, SM, pH), color parameters (MEXA, b*, ERYTH, ITA°), and texture parameters (R2, Ra) measured by a multifunctional skin physiology monitor to characterize the physiological status of Chinese women’s skin. The texture parameters (acne, acne marks, blackheads, pores), pigmentation parameters (total pigmentation, freckles, sunburn, age spots), and wrinkle parameters (total wrinkles, eyebrow lines, fine lines around the eyes, crow’s feet, and perioral lines) obtained from VISIA-CR image extraction were included to characterize the skin morphology status.

### Facial Skin Aging Rules Derived by Polynomial Fitting

With age, skin wrinkles increase, texture deteriorates, acne decreases, pigment spots increase, skin tone darkens, and sebum secretion decreases. Facial skin aging rules can find much support in histological studies on skin aging, which are largely consistent with previous findings. However, we calculated key time points based on correlation analysis and polynomial fit models. And key time points can provide resources for the precision of skin aging care.

There was no significant correlation between skin moisture content, pH, haem content and age. Crow’s feet began to appear on the face of the women around the age of 23. The first perioral wrinkles formed in most women around the age of 35. After 42 years of age, the number of skin wrinkles began to increase significantly (the fine lines around the eyes were extracted from the front image of the VISIA-CR; the area of the lateral canthus was small, so the fine lines around the eyes gave an error). From the age of 27 on, the skin was likely to have blackheads, and the number of pores increased continuously. The number of acne marks decreased with age. The roughness of the skin increased with age, fastest around age 37. Around age 47, the skin had the least elasticity and the most stains. The skin tone was the lightest around the age of 23 and then became darker continuously, reaching its fastest rate around the age of 41. The amount of sebum secretion decreased with age.

### Classification of the Four Aging Stages Based on Wrinkles

The skin parameters of the wrinkle dimension have the strongest correlations with age. In addition, the main characteristics of facial skin aging are wrinkles and sagging. Compared with other skin parameters, wrinkles more significantly affect appearance. Based on the wrinkle parameter–age polynomial fitting model, the analysis suggests that the number of wrinkles does not change significantly before the age of 30 but then begins to increase slowly, starts increasing rapidly around the age of 42, and increases steadily at a certain rate after the age of 47. Therefore, according to the above key age points, we divided the skin aging process into four stages: incubation period (aged 18–30), aging occurrence period (aged 31–42), rapid aging period (aged 43–47) and stable aging period (aged 48–60).

### Skin Characteristics Accompanying the Four Aging Stages

Different skin aging stages are accompanied by different skin characteristics. The incubation period is the critical period for the appearance of skin stains. The skin texture gradually deteriorates during the aging occurrence period. The rapid aging period is a critical period for the aging of skin parameters. Skin status is worst during the stable aging period. Based on the skin characteristics of different stages, researchers can develop skin care products suitable for different age groups in a targeted manner.

#### The Incubation Period (Aged 18–30) Is the Critical Period for the Appearance of Stains

**FIGURE 5:**
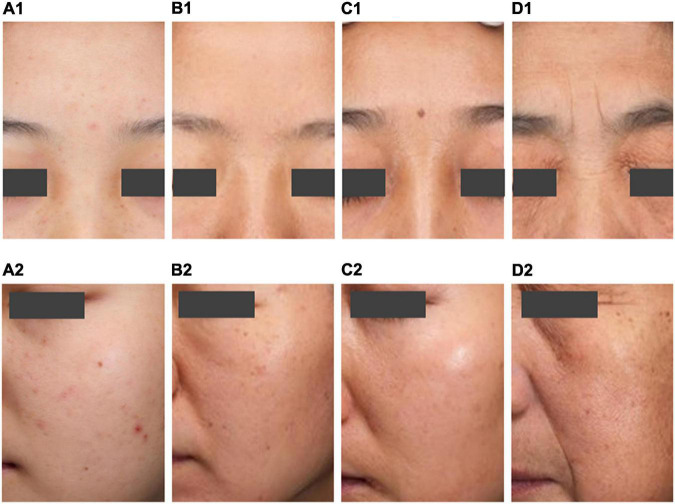
Typical map of the four aging stages. Typical map of the incubation period **(A1,A2)**. Typical map of the aging occurrence period **(B1,B2)**. Typical map of the rapid aging period **(C1,C2)**. Typical map of the stable aging period **(D1,D2)**.

Skin wrinkles and stains are at their lowest levels in the incubation period, but the incubation period is the key period for the formation and appearance of stains. The skin elasticity, under the texture dimension, is significantly higher, the number of blackheads and pores is significantly lower, and the number of acne marks is the largest. The melanin content and gloss, under the skin color dimension, are significantly lower, the skin color is light, and the yellowness is minimal. The sebum secretion is strong. Women in the incubation period have the best overall skin performance, but they may face skin problems such as acne, sunburn, poor barrier function, and oil. 1.1.1 (Figures 5A1,A2).

#### The Skin Texture Gradually Deteriorates During the Aging Occurrence Period (Aged 31–42)

Women in the aging occurrence period have fewer wrinkles. After entering the aging occurrence period, the skin texture gradually deteriorates, the skin roughness is significantly higher, the number of acne marks grows, and the pores are still few. The total stains and freckles are significantly more numerous than in the incubation period. This period has few age spots, less yellowish skin, and a light complexion. However, women in this period may face skin problems such as rough skin, acne marks, multiple spots, and poor barrier function (Figures 5B1,B2).

#### The Rapid Aging Period (Aged 43–47) Is a Critical Period for the Aging of Skin Parameters

The rapid aging period is a critical period when wrinkles and age spots increase, skin tone darkens, and skin texture changes. There are more total stains and freckles, the yellowness of the skin increases, the complexion darkens, and the secretion of sebum falls. In general, the rapid aging period is a critical period for changes in many skin parameters. Women in this period may face skin problems such as skin with more pigmented skin, dry skin, and less oily skin (Figures 5C1,C2).

#### Skin Status Is Worst During the Stable Aging Period (Aged 48–60)

During this period, women’s skin has the most wrinkles and stains. The skin texture is poor, characterized by low elasticity, high roughness, and the most blackheads and pores. The skin has significantly higher melanin content and gloss, the skin grows darker and yellower, and the secretion of sebum is the lowest. Women in the stable aging period have the most serious skin aging problems and may face dry and less oily skin (Figures 5D1,D2).

### Lateral Differences in Regional and Ethnic Skin Characteristics

Lateral differences in regional and ethnic skin characteristics have been widely demonstrated in women of different ages. Alexis et al. reviewed variations in skin barrier from literature studies and suggests racial/ethnic variations in ceramide content and filaggrin mutations. However, they also emphasized these studies frequently had methodological flaws, insufficient sample size, and demonstrated conflicting results (19). Experiments by Kompaore et al. suggest that TEWL measurements were higher in Asians and Blacks compared to Caucasians (20). Sebum secretion gradually decreased with age, which is consistent with previous literature conclusions of both Chinese (21, 22) and Indian female population (23). In a study about 834 female subjects each from a total of eight Asian cities in China, India, South Korea, Japan, and the Philippines grouped, characteristics related to skin surface sebum level decreased with age, which demonstrated common trends in skin characteristics among Asian populations (24). But sebum showed different results in the Caucasian population. Machková et al. studied skin parameters in 442 Caucasian women aged 23 to 63 years and found that sebum content increased until the age of 50 (2). Results of a Germany clinical study by S Luebberding et al. showed that whereas TEWL and stratum corneum hydration showed only very low correlation with subject’s age, the sebum production decreased significantly with age, resulting in the lowest skin surface lipids levels measured in subjects older than 70 years, which similar to our findings (25).

Asians are a population with various skin phototypes, ranging from type III to IV Fitzpatrick’s classification in Chinese and Japanese to type IV and V in Indian and Pakistani people (26). However, we found that the skin Fitzpatrick’s classification of Chinese women ranged from type I to type IV, with more types II and III in this study. This may be related to the fact that women have paid more attention to sun protection in recent years (27, 28). Pigmentary changes in Asians are more obvious problems relative to the earlier occurrence of wrinkles in Caucasians (29). In this study, we found the incubation period is the critical period for the appearance of skin stains (18–30 years old) of Chinese women. According to international practice, most studies of skin aging divide the groups into 5–10-year intervals, does not reflect skin age dynamic process. We proposed an unequal-distance aging division method based on the wrinkle parameters: incubation period (aged 18–30), aging occurrence period (aged 31–42), rapid aging period (aged 43–47) and stable aging period (aged 48–60), which was more in line with the dynamic aging rules. This grouping model can be used for grouping, volunteer recruitment, and nursing care in aging population studies.

### Longitudinal Differences in Skin Aging (Chronological Aging Versus Photoaging)

Skin aging is a complex biological behavior involving multiple skin components, which consists of chronological aging and extrinsic aging (30). chronological aging is determined by genetic factors, and with age, the skin gradually exhibits a programmed natural aging process, such as sagging and wrinkles. Histologically, chronological aging is mainly characterized by thinning of the epidermis and flattening of the dermis and epidermis junction (31). At the same time, a reduction in the surface area of the dermis and epidermis interface leads to atrophy of the skin’s dermis, a decrease in fibroblasts, and a decrease in subdermal adipose tissue. The number and diameter of collagen fiber bundles also decrease with age, and the ratio of type III collagen to type I collagen increases (32). Extrinsic aging is caused by living conditions such as diet and nutritional status (33), smoking (34), sleep (35), stress (36), and environmental factors such as air pollution (37) and solar radiation (38, 39). Among them, oxidative stress caused by ultraviolet radiation is considered to be the main cause of extrinsic skin aging, also known as photoaging (5). Clinical symptoms of photoaging include dryness, freckles, irregular pigmentation, loss of elasticity, and telangiectasia. In general, the chronological aging process is stable and the morphological and structural changes are uniform, while the photoaging shows more irregular morphological changes and even vegetations (40).

During aging, facial skin suffers from a combination of chronological aging and photoaging. This study aims to investigate the skin aging changes of Chinese women in cities, more than 98% of the volunteers recruited are engaged in indoor work activities, so their facial skin is not significantly affected by photoaging. Research by Zouboulis et al. found that histological features of photoaging include irregular hyperplasia of the sebaceous glands (41), whereas the volunteers in this study showed a marked decrease in skin oil content with age, the experimental conclusions are mutually confirmed. In addition, since the research objects are mainly affected by time chronological aging, the age of the volunteers is used as the independent variable instead of their skin aging speed, then the change trend of various skin parameters is observed through a polynomial fitting model. The speed of aging due to photoaging was not explored, which is one of the limitations of this study.

## Conclusion

**FIGURE 6:**
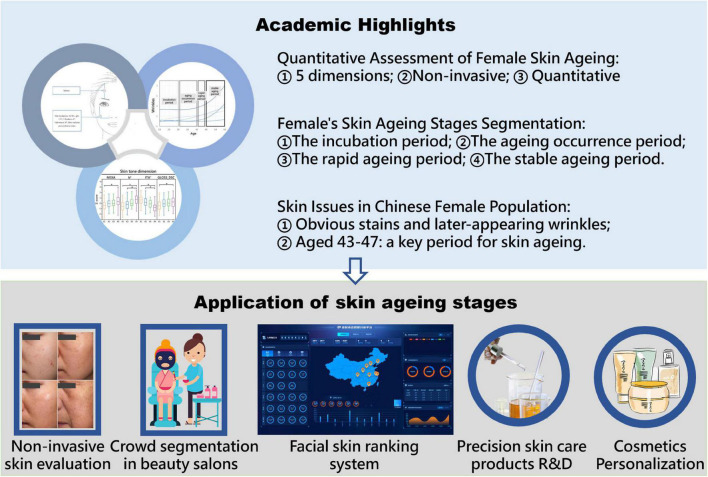
The academic value and application orientation of this research.

Personalized skincare has become a hot topic in dermatology and cosmetics field in recent years. Bonati et al. recommend age-appropriate cosmetic preventions and interventions (42). One of the foundations for personalization is the accurate quantitative measurement of facial skin by utilize the physiological characteristics. A comprehensive evaluation system to describe facial skin should be done from two aspects: physiological status and morphological status. The physiological status of female skin is closely related to health, such as a lower melanin content associated with an increased prevalence of skin cancer (43). The morphological characteristics of the skin are closely related to skin aging, such as wrinkles and sagging (9). In this study, we aim to a comprehensive scientific categorization by characterized the facial skin status from five dimensions: skin barrier, color, texture, stains, and wrinkles. This model can be extended to dermatology. Multidimensional profiling based on non-invasive test can be used to systematically and quantitatively study the apparent characteristics of skin aging. Moreover, different skin aging stages are accompanied by various skin characteristics. Based on this work, the skin characteristics of different stages, researchers can develop skin care protocol and products suitable for people in different age stage (Figure 6).

## Data Availability Statement

The raw data supporting the conclusions of this article will be made available by the authors, without undue reservation.

## Ethics Statement

The studies involving human participants were reviewed and approved by the Ethics Committee of Xiyuan Hospital of China Academy of Chinese Medical Sciences (2019XL013-2) and was registered in the Chinese Clinical Trial Registry (registration number: ChiCTR1900025405). The patients/participants provided their written informed consent to participate in this study. Written informed consent was obtained from the participants for the publication of any potentially identifiable images or data included in this article.

## Author Contributions

X-XY was in charge of the first draft and data analysis of the article. FY instructed the method construction of the study. M-MZ and Y-FH participated in the selection of statistical methods. HM participated in the methodological development of this study and interpreted the findings on skin. Q-YM clarified the research direction. Q-YS refined the specific research content. All authors contributed to the article and approved the submitted version.

## Conflict of Interest

Q-YM and Q-YS were employed by the Shanghai Pechoin Daily Chemical Co., Ltd. The remaining authors declare that the research was conducted in the absence of any commercial or financial relationships that could be construed as a potential conflict of interest.

## Publisher’s Note

All claims expressed in this article are solely those of the authors and do not necessarily represent those of their affiliated organizations, or those of the publisher, the editors and the reviewers. Any product that may be evaluated in this article, or claim that may be made by its manufacturer, is not guaranteed or endorsed by the publisher.
