# Supplementary material for: Facial Skin Aging Stages in Chinese Females
Source: Front Med (Lausanne). 2022 Apr 27;9:870926. doi: 10.3389/fmed.2022.870926 (PMC9094048; doi:10.3389/fmed.2022.870926)
Supplement: Supplementary file 1 [file Data_Sheet_1.PDF]

Supplementary Material

1 Supplementary Data

Supplementary Materials provides 20 skin parameter-age polynomial fit models.

1.1 Wrinkle dimension

The skin parameters of wrinkle dimension show an overall upward trend; crow's feet and perioral lines increase with age, crow's feet appear around 23 years old, and perioral lines increase at the fastest rate around 35 years old. Total wrinkles, eyebrow lines and fine lines around the eyes increased significantly after the age of 42, and the growth rate was getting faster and faster.

Total wrinkles (Figure S1A)

$$\boldsymbol{f(x) = 0.0001352x^3 - 0.01068x^2 + 0.2712x + 0.1146}$$

With age, the total wrinkles show a trend of stable first and then rise; the total number of wrinkles is relatively stable before the age of 31 with an average of about 2; wrinkles begin to increase slowly with age between 31-40 years old; after 40 years of age, the total number of wrinkles increases rapidly.

Eyebrow lines (Figure S1B)

$$\boldsymbol{f(x) = 0.0001482x^3 - 0.01337x^2 + 0.3857x - 3.456}$$

After 36 years of age, eyebrow lines appear and show an overall upward trend with age; in the age group of 36-45 years, the eyebrow lines gradually increased with age; the number of eyebrow lines increased rapidly after the age of 45.

Fine lines around the eyes (Figure S1C)

$$\boldsymbol{f(x) = 0.00002764x^3 - 0.00284x^2 + 0.09259x - 0.9496}$$

The fine lines around the eyes appear after 42 years of age and show an overall upward trend with age; in the age group of 42-50 years, fine lines around the eyes slowly increase with age; after the age of 50, the number of fine lines around the eyes increases rapidly.

Crow's feet (Figure S1D)

$$\boldsymbol{f(x) = -0.00002792x^3 + 0.004999x^2 - 0.1865x + 2.032}$$

As age grows, the overall crow's feet show a continuous upward trend. After the age of 30, the number of crow's feet increases at a relatively rapid rate. It is estimated that the number of crow's feet will increase the fastest around the age of 60.

Perioral lines (Figure S1E)

$$f(x) = 0.00003081x^3 - 0.003278x^2 + 0.146x - 1.509$$

Perioral lines gradually appear after the age of 18, and show an overall upward trend with age; around 35 years old, the rate of increase in perioral wrinkles reaches the fastest.

1.2 Texture dimension

With age, the number of blackheads and pores decreases first and then continues to rise. After 27 years of age, blackheads begin to increase, and the rate of increase of blackheads is the fastest around 52 years of age. After 29 years of age, the number of pores begins to increase, and the rate of increase of pores is the fastest around 50 years of age; the skin roughness Ra shows an upward trend, and the skin roughness Ra increases the fastest around 37 years old; the elasticity R2 shows a trend of first decline and then slowly rises, and the skin elasticity is the smallest around 47 years old; the number of acne and pimples decreases with age.

R2 (Figure S1F)

$$f(x) = 0.000001739x^3 - 0.00006185x^2 - 0.005867x + 0.8063$$

With age, the R2 value of skin elasticity decreases first and then rises slowly; the older the age, the worse the elasticity, and the smallest skin elasticity is around 47 years old.

Ra (Figure S1G)

$$f(x) = 0.0000004386x^3 - 0.00004842x^2 + 0.001855x - 0.001362$$

With age, the overall Ra value of skin roughness shows an upward trend, and the growth rate of skin roughness is the fastest around 37 years old; the older the age, the rougher the skin.

Acne (Figure S1H)

$$f(x) = -0.0005298x^3 + 0.06741x^2 - 2.857x + 53.26$$

With age, acne shows a trend of first decline, then slowly rise and then decline; after 41 years of age, the number of acne starts to increase slowly; after 44 years of age, the number of acne continues to decrease.

Acne marks (Figure S1I)

$$f(x) = -0.0001441x^3 + 0.01685x^2 - 0.8035x + 20.6$$

With age, the number of acne marks shows a downward trend, and the rate of decline is the fastest around the age of 39; the older the age, the fewer skin acne marks.

Blackheads (Figure S1J)

$$f(x) = -0.0002073x^3 + 0.03228x^2 - 1.295x + 19.32$$

The number of blackheads first declines and then rises; the number of blackheads is the smallest around 27 years of age; after that, the number of blackheads increases, with the largest growth rate of blackheads around the age of 52; it is estimated that the number of blackheads is the largest around the age of 77.

Pores (Figure S1K)

$$\boldsymbol{f(x) = -0.001265x^3 + 0.19x^2 - 7.765x + 129.7}$$

The number of pores first declines and then rises with age; the number of skin pores is the smallest around 29 years old, then the pores begin to increase, and the growth rate of pores is the largest around 50 years old; it is estimated that the number of pores is the largest around 72 years old.

1.3 Skin stain dimension

As age increases, the total stains and freckles first increase and then decrease, reaching the maximum at about 48 years old; the overall age spots are on the rise. After 28 years old, the number of age spots increases significantly, and the growth rate continues to accelerate; the number of sun spots is first After falling and then rising, the number of sunburns is the smallest around the age of 25, and then begins to increase. The growth rate is the largest around the age of 40, and the number of sunburns is the largest around the age of 54.

Total stain (Figure S1L)

$$\boldsymbol{f(x) = -0.002859x^3 + 0.2672x^2 - 5.792x + 88.32}$$

The total number of stains first increases and then decreases with age; the growth rate of stains is the largest around the age of 31, and the total number of stains is the largest around the age of 48; the decline rate of total stains increases constantly after the age of 48.

Freckles (Figure S1M)

$$\boldsymbol{f(x) = -0.002534x^3 + 0.2272x^2 - 4.389x + 72.35}$$

The number of freckles first increases and then decreases with age; the growth rate of freckles is the largest around the age of 30, and the number of freckles is the largest around the age of 48; the decline rate of total freckles increases constantly after the age of 48.

Sunburns (Figure S1N)

$$\boldsymbol{f(x) = -0.000315x^3 + 0.03751x^2 - 1.283x + 14.45}$$

The number of sunburns first decreases, then increases, and then declines with age; the number of sunburns is the least at 25 years old; after that the number of sunburns increases, with the highest growth rate around the age of 40, and the largest number of sunburns around the age of 54.

Age spots (Figure S1O)

$$\boldsymbol{f(x) = -0.000009078x^3 + 0.002513x^2 - 0.1205x + 1.52}$$

With age, the number of age spots shows an upward trend. The number of age spots decreases slowly before the age of 28, but the trend is not obvious; after the age of 28, the number of age spots increases significantly, and the rising rate continues to increase; the older the age, the more age spots.

1.4 Skin tone dimension

The GLOSS_DSC value of skin gloss and the MEXA value of skin melanin content showed an upward trend, increasing consistently with age. The skin yellowness b* value showed a trend of first decline and then increase. The skin yellowness was the smallest around 26 years old, and then continued to increase. The growth rate was the largest around 43 years old, and the yellowest skin around 60 years old. The ITA° value first rises, then decreases, and then rises. The skin tone is the lightest around the age of 23, and then the skin tone becomes darker. The skin tone darkening rate is the largest around the age of 41, and the ITA° value of the skin around the age of 59 is the smallest, and the skin tone is the darkest.

MEXA (Figure S1P)

$$\boldsymbol{f(x) = 0.0001623x^3 + 0.0001347x^2 - 0.05713x + 132.5}$$

With age, the overall MEXA value of skin melanin content is increasing, and the growth rate is getting faster and faster; the older the age, the higher the MEXA value of skin melanin content.

b* (Figure S1Q)

$$\boldsymbol{f(x) = -0.000197x^3 + 0.02533x^2 - 0.9183x + 21.56}$$

As age increases, the b* value of skin yellowness decreases first, then rises, and then decreases; the b* value decreases constantly, reaching the minimum at 26 years old; after that, the yellowness increases constantly, and the growth rate is the largest around 43 years old, the b* value is highest around 60 years old, this is when the skin is the yellowest.

ITA° (Figure S1R)

$$\boldsymbol{f(x) = 0.0006319x^3 - 0.07765x^2 + 2.585x + 21.06}$$

As age increases, the ITA° value first rises, then decreases, and then rises; ITA° value continues to increase, with the lightest skin tone around 23 years old; after that, the skin tone gets darker and darker at around 41 years old; the skin tone darkens the most at around 59 years old. The ITA° value is the smallest and the skin tone is the darkest.

GLOSS_DSC (Figure S1S)

$$\boldsymbol{f(x) = -0.0000126x^3 + 0.00142x^2 - 0.02532x + 3.795}$$

The GLOSS_DSC value of skin gloss increases with age; the growth rate is the largest around 38 years old, and the GLOSS_DSC value of skin gloss is expected to be the largest around 65 years old.

1.5 Barrier dimension

The TEWL value of transdermal water loss increased first, then decreased, and then increased with age. The TEWL was the largest at about 30 years old, the TEWL decreased the fastest at about 43 years old, and the TEWL value was the smallest at about 57 years old; with age, the amount of sebum secretion SM The value shows a downward trend, the older the age, the lower the sebum secretion.

TEWL (discard)

$$f(x) = 0.0002636x^3 - 0.03437x^2 + 1.343x + 0.563$$

With age, the TEWL value of transcutaneous water loss showed a trend of rising first, then falling, and then slowly rising (almost unchanged). The analysis by Reva P. Peer et al. found that due to uncontrolled experimental variables, the repeatability of TEWL was difficult to guarantee. Therefore, we discarded this skin parameter.

SM (Figure S1T)

$$f(x) = -0.0008836x^3 + 0.1043x^2 - 5.048x + 132.9$$

The overall SM value of sebum secretion showed a downward trend with age; the older the age, the less sebum secretion.

2 Supplementary Figures

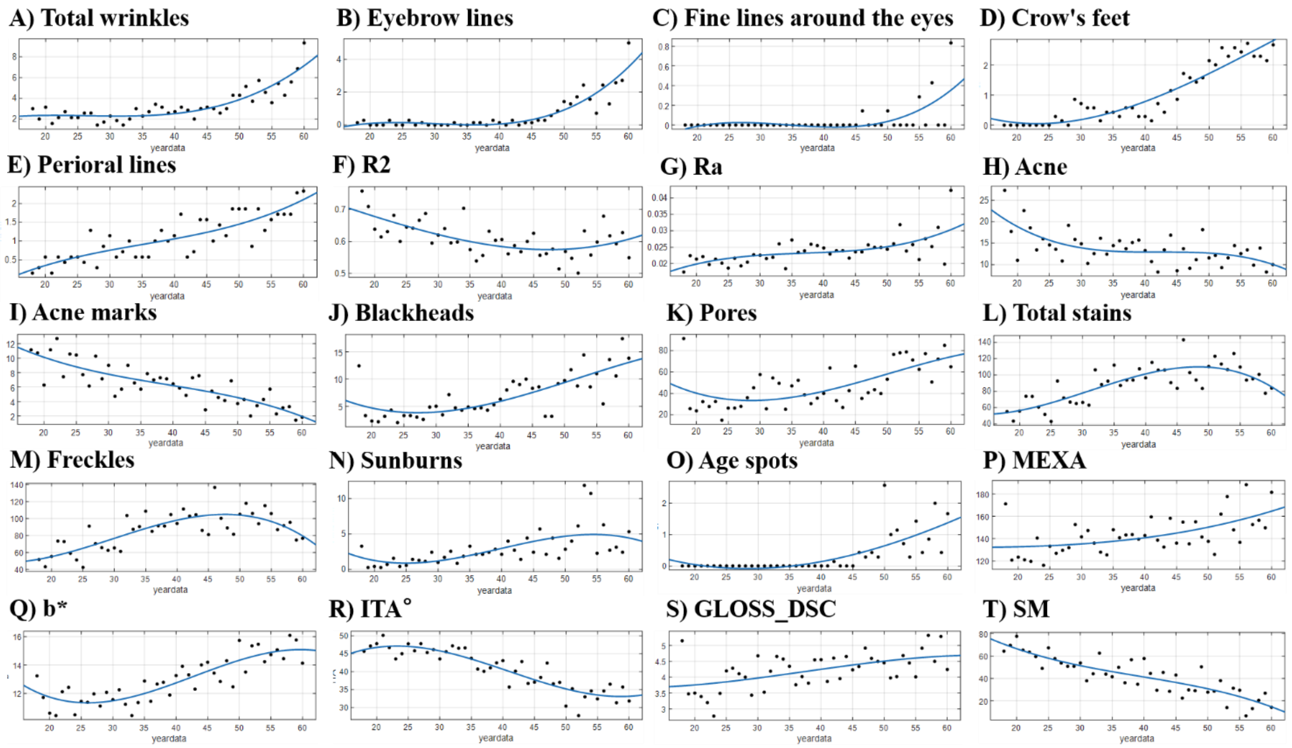

Supplementary Figure 1. Skin parameters-age polynomial fitting models.
